# Accurate and Efficient KIR Gene and Haplotype Inference From Genome Sequencing Reads With Novel K-mer Signatures

**DOI:** 10.3389/fimmu.2020.583013

**Published:** 2020-11-26

**Authors:** David Roe, Rui Kuang

**Affiliations:** ^1^ Bioinformatics and Computational Biology, University of Minnesota, Rochester, MN, United States; ^2^ Department of Computer Science and Engineering, University of Minnesota, Minneapolis, MN, United States

**Keywords:** killer-cell immunoglobulin-like receptor, genotype, haplotype, interpretation, natural killer, whole genome sequencing (WGS)

## Abstract

The killer-cell immunoglobulin-like receptor (KIR) proteins evolve to fight viruses and mediate the body’s reaction to pregnancy. These roles provide selection pressure for variation at both the structural/haplotype and base/allele levels. At the same time, the genes have evolved relatively recently by tandem duplication and therefore exhibit very high sequence similarity over thousands of bases. These variation-homology patterns make it impossible to interpret KIR haplotypes from abundant short-read genome sequencing data at population scale using existing methods. Here, we developed an efficient computational approach for *in silico* KIR probe interpretation (KPI) to accurately interpret individual’s KIR genes and haplotype-pairs from KIR sequencing reads. We designed synthetic 25-base sequence probes by analyzing previously reported haplotype sequences, and we developed a bioinformatics pipeline to interpret the probes in the context of 16 KIR genes and 16 haplotype structures. We demonstrated its accuracy on a synthetic data set as well as a real whole genome sequences from 748 individuals from The Genome of the Netherlands (GoNL). The GoNL predictions were compared with predictions from SNP-based predictions. Our results show 100% accuracy rate for the synthetic tests and a 99.6% family-consistency rate in the GoNL tests. Agreement with the SNP-based calls on KIR genes ranges from 72%–100% with a mean of 92%; most differences occur in genes *KIR2DS2*, *KIR2DL2*, *KIR2DS3*, and *KIR2DL5* where KPI predicts presence and the SNP-based interpretation predicts absence. Overall, the evidence suggests that KPI’s accuracy is 97% or greater for both KIR gene and haplotype-pair predictions, and the presence/absence genotyping leads to ambiguous haplotype-pair predictions with 16 reference KIR haplotype structures. KPI is free, open, and easily executable as a Nextflow workflow supported by a Docker environment at https://github.com/droeatumn/kpi.

## Introduction

Human chromosome 19q13.4 contains a ~150–250 kilobase region encoding 16 genes of the natural killer-cell immunoglobulin-like receptor (KIR) family. These genes are ~4–16 kilobases long and evolved *via* tandem duplication during primate evolution (1, 2). The KIR receptors recognize human leukocyte antigen (HLA) class I molecules and contribute to natural killer (NK) cell functions *via* activating or inhibiting signals. These receptor-ligand pairs coevolved under selection pressures from reproduction and pathogenic defense (3), and it is believed that KIR genes have undergone a balancing selection *via* duplications and deletions into two broad categories of haplotypes, in which one category tends to vary more at the allelic level and the other tends to vary more at the structural (gene content and order) level (4–6). A few dozen KIR full haplotype sequences and approximately 2500 full- or inter-gene sequences have been publicly deposited (5, 7, 8). Haplotype structures are divided into two classes (9). Class ‘A’ contains one haplotype and its deleted forms. Class ‘B’ haplotypes are more structurally diverse and contain a variety of insertions and deletions. Generally, the A haplotype occurs with 50-60% frequency, haplotypes that are half-A and half-B occur with 30-40%, and the rest of the haplotypes are variants of the B haplotypes. Except for some rarer deleted forms, KIR haplotypes are structurally variable around 4 ‘framework’ genes (*KIR3DL3*, *KIR3DP1*, *KIR2DL4*, *KIR3DL2*), with *KIR3DL3* through *KIR3DP1* defining the proximal (or ‘centromeric’) region and *KIR2DL4* through *KIR3DL2* defining the distal (or ‘telomeric’) region, with the two gene-rich regions separated by the relatively large and recombinant *KIR3DP1-KIR2DL4* intergene region.

It is difficult to interpret the KIR region with high-throughput sequencing reads for an individual human genome when the structural arrangements are unknown; indeed, it is difficult even when the structural haplotypes are known, since the read length is too short to map unambiguously to the repetitive and homologous KIR genes. As a consequence, the reads from KIR region are ignored, as to the best of our knowledge, there are currently no algorithms to interpret KIR from whole genome sequencing (WGS). SNP (single nucleotide polymorphism)-based KIR interpretation is more commonly applied. For example, KIR*IMP is a web-application to predict genes and haplotypes from microarray SNP genotypes (10). As an algorithm whose raw data is microarray calls, KIR*IMP can interpret KIR from genome wide SNP arrays, but it is not applicable to interpret KIR from raw sequences.

Since a general solution for KIR structural interpretation from raw genomic DNA is not currently available, this study implements such an algorithm for the prediction of KIR genes and full structural haplotypes from any type of raw full-region-or-greater genomic sequence at population scale. In particular, we systematically evaluated small markers for KIR genes and then applied those markers to a synthetic KIR probe interpretation (KPI) algorithm for the presence/absence of 16 KIR genes and 16 haplotype structures. Our approach leverages recent bioinformatics innovations for short sequence (‘probe’) genotyping, along recently published KIR reference haplotypes. The KPI algorithm first efficiently counts the occurrence of each kmer probe in the raw sequences, and then uses multiple probes per gene to call its presence/absence. Those 16 genotypes are then used to generate haplotype-pair predictions. In the experiments, we report 100% accuracy on a test set of synthetic haplotypes for comparisons with known truth. We also report that gene and haplotype-pair predictions for the WGS GoNL cohort are family consistent and compare favorably with reference frequencies in comparison to SNP-based predictions using KIR*IMP.

## Materials and Methods

### Overview

The workflow of KPI consists of three steps,

1. Discover the 25mer gene markers based on a multiple sequence alignment analysis of 68 full-length haplotype sequences.
2. Count the 25mer markers in the reads of genomic DNA per individual to generate the individual’s 25mer genotype.
3. Predict presence/absence per gene from the marker genotypes for each individual.
4. Predict haplotype pairs from the gene presence/absence calls for each individual.

In the following, we first explain each step and then describe the synthetic data and GoNL data used for the evaluation.

### Step 1: Discovering 25mer Gene Markers

To discover gene marker 25mers, first a multiple sequence alignment (MSA) was created with 68 publicly deposited full-length haplotypes sequences (11). Briefly, each haplotype was annotated at an average resolution of ~4kbp using a set of 15 120-base markers. This high-level annotation was aligned into a MSA representing a structural alignment of all haplotypes. Then, each subregion was aligned to base pair resolution. This resulted in a full resolution, full haplotype MSA that accurately classifies each allele into a haplotype-defined locus, and it aligns the alleles precisely at each locus. The haplotype and gene annotations of the MSA provided a list of full-length alleles for 16 genes: *KIR2DL1-5, KIR2DS1-5, KIR2DP1, KIR3DL1-3, KIR3DP1*, and *KIR3DS1*. Markers for each gene locus were chosen by selecting all sequences of length 25 (25mers) present in every allele of the gene but not elsewhere in the KIR haplotypes nor the rest of the genome reference GRCh38. More details about the algorithm are in **Supplemental Figure 1**. The marker sequences are in **Supplemental Data Sheet 1** and also checked in to GitHub at https://github.com/droeatumn/kpi/tree/master/input in text and fasta format.

### Step 2: Count 25mer Markers

KMC 3, with workflows implemented in Nextflow (12) and Apache Groovy (13) and a software environment implemented as a Docker container, is used to create 25mer databases from sequence or short-read data and match the markers across the datasets. Using KMC 3, we generate the list of all 25mers from the short reads of each individual and then match the 25mers in the marker databases to report the hit counts of each 25mer marker in the individual. Details are in **Supplemental Figure 1**.

### Step 3: Individual Genotyping From 25mer Markers

KPI calls presence/absence per gene by aggregating the presence/absence genotypes of many small (25mer) markers, each specific to one gene. 25mers with hit counts less than three are considered sequencing errors and set to zero. If the mean hit count of all the markers per gene is zero, then the gene is predicted absent; otherwise, it is called present. Additional details can be found in **Supplemental Figure 1**.

### Step 4: Individual Haplotyping From Genotypes

**Figure 1:**
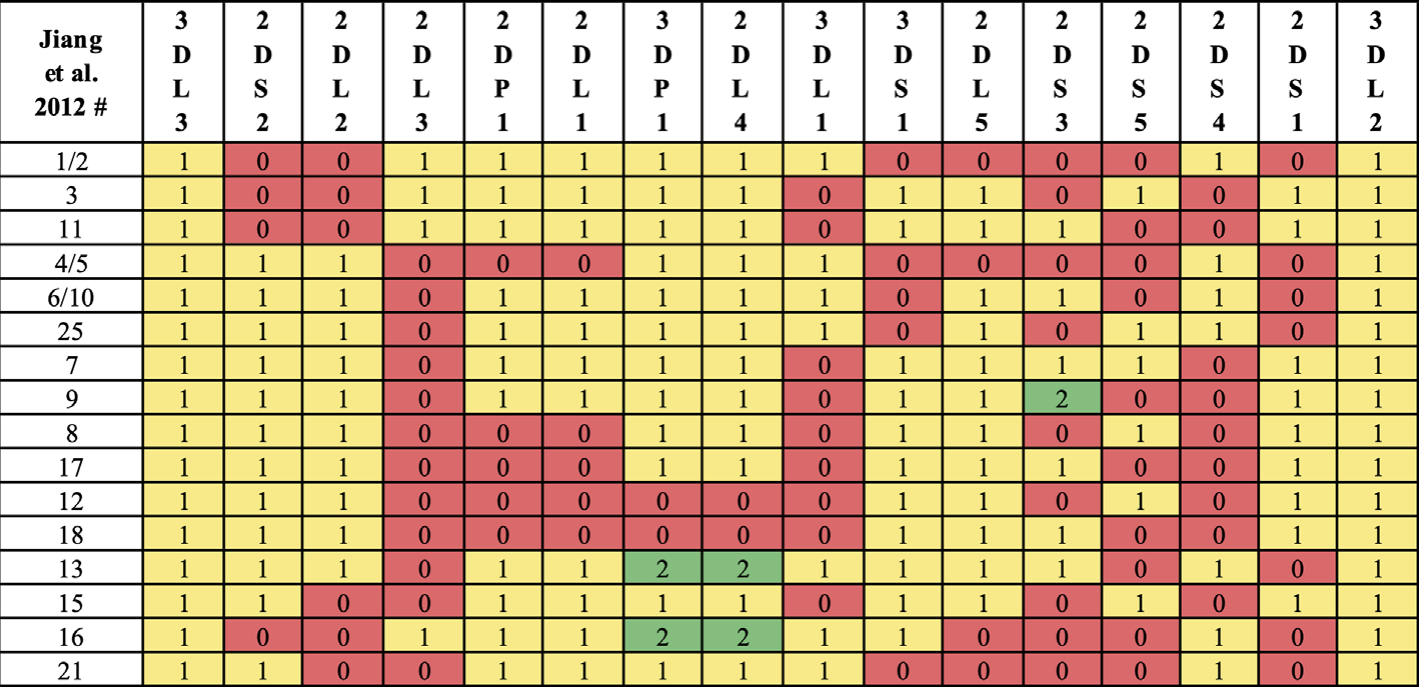
Reference haplotype definitions. Haplotype numeric labels (Jiang et al. 2012) are shown with their definition *via* gene counts. Following Jiang et al. convention, some haplotypes (e.g., 7, 9) are distinguished by *KIR2DS3*/*KIR2DS5* alleles instead of structural differences. In this study, some haplotypes (e.g., 1, 2) are combined, as *KIR2DS4* full/deleted alleles are not considered in KPI’s genotyping.

Haplotype-pair predictions were made by fitting the genotype to all possible pairs of the 16 structural reference haplotypes defined in **Figure 1**. The numbers and frequencies of the haplotypes are from Jiang et al. 2012 (4) (**Table 1**); some of their haplotypes are combined because Jiang et al. consider certain alleles as separate haplotypes, such as full or deleted alleles of *KIR2DS4*. These 16 haplotypes represent 97% of all haplotypes in the Jiang et al. report.

**Table 1:** Reference haplotype names and frequencies.

Jiang et al. 2012 #	informal names	Jiang et al. 2012 freq.
1/2	cA01~tA01	55.2%
3	cA01~tB01_2DS5	10.9%
11	cA01~tB01_2DS3	1.4%
4/5	cB02~tA01	12.8%
6/10	cB01~tA01_2DS3	6.9%
25	cB01~tA01_2DS5	0.1%
7	cB01~tB01_2DS3_2DS5	2.6%
9	cB01~tB01_2DS3_2DS3	2.1%
8	cB02~tB01_2DS5	2.1%
17	cB02~tB01_2DS3	0.3%
12	cB04~tB03_2DS5	0.8%
18	cB04~tB03_2DS3	0.3%
13	cB01~tB05	0.7%
15	cB05~tB01	0.4%
16	cA01~tB05	0.3%
21	cB05~tA01	0.2%
sum		97.0%

The first column contains the numeric label assigned to haplotypes in Jiang et al. (2012). Column 2 contains the informal names along with a reference frequency in column 3.

For the GoNL predictions, haplotype ambiguity was reduced by family trio patterns and then further by the EM (Expectation-Maximization)-based methods as described and used in Vierra-Green 2012 (14). Haplotype frequencies were calculated from the EM-reduced individual haplotype-pair predictions. These haplotype frequency calculations are not possible on the KPI’s haplotype-pair predictions because they can be ambiguous.

### Synthetic Capture on Diploid Data

KPI was evaluated on a synthetic test set. There are six reference haplotype structures with publicly deposited full-length sequences (**Figure 1**, top six rows). For each of these six structures, one sequence was randomly chosen to represent that structure, and it was paired with a random haplotype sequence from the set of all sequences, with an equal probability for each sequence. dwgsim (12) was used to generate 10,000 2×150 pair reads per haplotype (~20× coverage) with 1% error rate. This provided a simulated six-person validation set of six diploid whole-region short-read sequences, representing all fully sequenced haplotype structures and paired to provide a variety of genotypes. The sequences are included in **Supplemental Data Sheet 2**.

### GoNL Family WGS and Immunochip SNP Data

KPI was also run on a large real-world example. WGS was obtained from The Genome of the Netherlands (GoNL) (13), a genome sequencing project whose goal is to map the genetic variation within the population of Netherlands in 250 family trios (750 individuals). The project provided non-paired sequencing of the whole genomes of the population, which was done on the Illumina HiSeq 2000 platform. Coverage of the KIR region were similar to the previously reported (13) whole-genome average of ~10–15×. Two individuals from two different families were removed from the GoNL project for data quality reasons, giving a total of 748 individuals.

KPI’s GoNL predictions were compared with results from microarray-based interpretation algorithm KIR*IMP. Illumina Immunochip microarray SNP data was obtained from GoNL (13). The data was prepared and executed following instructions using KIR*IMP v1.2.0 on 2019-10-05.

To the best of our knowledge, there only exists one method to predict KIR gene content from WGS sequences (15). However, we were unable to obtain results with it for both evaluation data sets. According to the authors, the current version is deprecated and to be replaced soon (16).

## Results

The predictions were evaluated in the small synthetic test set, where truth is known and a large real-world test set, where truth is unknown except for family relationships. Predictions were evaluated by comparing gene and haplotype-pair predictions to: known truth in the synthetic cohort, and family consistency (real-world cohort only), reference frequencies from Jiang et al.’s family copy number study (4), and the Allele Frequencies Database (17) in the real-world cohort. The real-world cohort was also compared with predictions from microarray-based algorithm KIR*IMP, although KIR*IMP was not considered ground truth as it reports accuracies as low as 81% for some genes (10). Haplotype-pair predictions were considered to be family consistent if each parents’ two haplotype predictions contained at least one of the child’s two predictions and one of the child’s haplotypes occurred in one parent and the other haplotype occurred in the other parent.

### Synthetic Evaluation

**Table 2** shows the results of the synthetic tests. The gene present/absent calls were 100% accurate for all genes. Although the haplotype predictions are ambiguous in half of the individuals, all are consistent with the ground truth.

**Table 2:** Results of synthetic tests.

haplotype 1 structure	haplotype 2 structure	haplotype 1 GenBank accession	haplotype 2 GenBank accession	KPI haplotype prediction	haplotypes consistent w/ truth?	gene prediction accuracy
cA01~tA01	cA01~tA01	GU182344	GU182340	cA01~tA01+cA01~tA01	Y	100%
cA01~tB01	cA01~tA01	KU645197	GU182360	cA01~tA01+cA01~tB01 or cA01~tB01+cA01~tB05	Y	100%
cB01~tA01	cA01~tA01	GU182351	NC000019.10	cA01~tA01+cB01~tA01	Y	100%
cB01~tB01	cB02~tA01	GU182339	GU182353	10 possibilities, including cB01~tB01+cB02~tA01	Y	100%
cB02~tA01	cA01~tA01	GU182341	KP420442	cA01~tA01+cB02~tA01	Y	100%
cB02~tB01	cA01~tA01	GU182359	KP420439	9 possibilities, including cA01~tA01+cB02~tB01	Y	100%

The first four columns detail the sequences from which the tests (n=6 haplotype-pairs) were generated. The fifth column is killer-cell immunoglobulin-like receptor probe interpretation’s (KPI’s) haplotype predictions, some of which are summarized for display.

### GoNL Evaluation

**Table 3** shows a summary of the gene prediction results from KPI and KIR*IMP on the GoNL data set. A reference frequency range is included from Allele Frequencies Net, selecting European cohorts >= 500 individuals. Overall agreement between KIR*IMP and KPI for the 16 genes (**Table 3**, column 6) ranges from 72% to 100%, with a mean of 92%. KIR*IMP differs from the reference haplotype (**Table 3**, column 7) frequency range by >10% in four genes (*KIR2DS2*, *KIR2DL2*, *KIR2DL5*, and *KIR2DS3*) compared with 0 genes for KPI (**Table 3**, column 8). Both KIR*IMP and KPI differ from the *KIR2DS1* reference by 9-10%, although the two algorithms agree in 98% of individuals for that gene.

**Table 3:** Summary of killer-cell immunoglobulin-like receptor (KIR)*IMP and KIR probe interpretation (KPI) gene predictions.

gene	reference freq.	KIR*IMP freq.	KPI freq.	KIR*IMP - KPI	KIR*IMP & KPI agreement	KIR*IMP - reference	KPI - reference
2DS2	53-54%	39%	52%	−13%	**72%**	**−14%**	**−1%**
2DL2	53-54%	39%	51%	−12%	**72%**	**−14%**	**−2%**
2DL3	90%	96%	92%	4%	92%	6%	2%
2DP1	96%	99%	97%	2%	97%	3%	1%
2DL1	96%	99%	97%	2%	97%	3%	1%
3DP1	100%	100%	100%	0%	100%	0%	0%
2DL4	100%	100%	100%	0%	100%	0%	0%
3DL1	93%–94%	96%	96%	0%	100%	2%	2%
3DS1	38%–44%	33%	35%	−2%	97%	−5%	−3%
2DL5	53%–56%	38%	47%	−9%	**87%**	**−15%**	**−6%**
2DS3	30%–31%	10%	29%	−18%	**81%**	**−20%**	**−1%**
2DS5	30%–36%	25%	27%	−2%	96%	−5%	−3%
2DS4	92%–94%	96%	96%	0%	100%	2%	2%
2DS1	43%–44%	33%	34%	−1%	98%	−10%	−9%
average					92%		

Frequencies relative to Genome of the Netherlands (GoNL) cohort of 748 individuals. The abbreviated gene name is in column 1. Column 2 lists the reference frequencies from The Allele Frequency Net Database. The predicted frequencies from KIR*IMP and KPI are in columns 3 and 4, respectively. The delta between KIR*IMP and KPI is shown in the column 5. Column 6 shows the agreement between KIR*IMP and KPI. Column 7 shows the delta between KIR*IMP and the reference. Column 8 shows the delta between KPI and the reference. Frequencies with differences >10% are in bold.

**Table 4** breaks down the differences between KIR*IMP and KPI in a confusion matrix. In the cases where KIR*IMP calls present (‘P’) and KPI calls absent (‘A’) (**Table 4**, column 2), the largest discrepancies are found in the centromeric genes *KIR2DS2* (8%), *KIR2DL2* (8%), and *KIR2DL3* (6%). In the reverse cases, when KIR*IMP calls absent and KPI calls present (**Table 4**, column 3), the largest discrepancies are greater and occur with the centromeric *KIR2DS2* (20%), *KIR2DL2* (20%), and the paralogous (centromeric or telomeric) *KIR2DL5* (11%), and *KIR2DS3* (19%).

**Table 4:** Confusion matrix of killer-cell immunoglobulin-like receptor (KIR)*IMP and KIR probe interpretation (KPI) gene predictions.

gene	KIR*IMP:P KPI:A	KIR*IMP:A KPI:P	KIR*IMP:P KPI:P	KIR*IMP:A KPI:A
2DS2	8%	**20%**	32%	40%
2DL2	8%	**20%**	31%	41%
2DL3	6%	2%	90%	2%
2DP1	2%	0%	97%	0%
2DL1	2%	1%	97%	0%
3DP1	0%	0%	100%	0%
2DL4	0%	0%	100%	0%
3DL1	0%	0%	96%	4%
3DS1	1%	3%	32%	64%
2DL5	2%	**11%**	36%	51%
2DS3	1%	**19%**	10%	71%
2DS5	1%	3%	24%	72%
2DS4	0%	0%	96%	4%
2DS1	1%	1%	33%	66%

Frequencies relative to GoNL cohort size of 748 individuals. The abbreviated gene names are in column 1. Column 2 lists the cases when KIR*IMP calls present (‘P’) and KPI calls absent (‘A’). Column 3 lists the cases when KIR*IMP calls absent (‘A’) and KPI calls present (‘P’). Column 4 is when they both call present. Column 5 is when they both call absent. Discrepancies >10% are in bold.

Per-individual haplotype-pair predictions for the GoNL cohort are included in **Supplemental Table 1**. Three lists of haplotype-pairs are provided: one for the initial fitting of all possible haplotype-pairs that could explain the genotype (i.e., KPI’s output); another that reduces those possibilities by family relationships; and one for the EM-reduced final haplotype-pair predictions.

KIR*IMP makes one most-likely prediction for all individuals. KPI’s predictions are sometimes ambiguous, with most predictions (mode) having one haplotype-pair but a mean of 2.3, standard deviation of 2.5, and a maximum of 14 haplotype-pair predictions per individual in the context of the 16 reference haplotypes. The KIR*IMP predictions are family consistent 100% of the time compared with 99.6% for KPI. However, the haplotype pair predictions between the two algorithms are concordant only 58% of the predictions.

**Table 5** compares the haplotype predictions between KIR*IMP and the EM-reduced haplotype pair predictions from the KPI output. KIR*IMP fit 100% of its predicted genotypes into 15 of its reference haplotypes. KPI fit 97% of its predicted genotypes into its 16 reference haplotypes. KIR*IMP made predictions for two haplotypes (cA01~tB04 and cB04~tB03, numbered 14, 18, and 12), totaling 0.47%, that are not in KPI’s set of reference haplotypes. KPI’s haplotype-pair predictions are too ambiguous to summarize in haplotype frequencies.

**Table 5:** Comparison of killer-cell immunoglobulin-like receptor (KIR)*IMP highest probability and EM-reduced haplotype prediction frequencies.

hap	reference frequency	KIR*IMP	KPI w/ EM	KIR*IMP -	KPI w/ EM -	KIR*IMP -
#				reference	reference	KPI w/ EM
1	55.20%	71.86%	59.70%	16.70%	4.50%	12.17%
2						
3	10.90%	12.57%	9.60%	1.70%	−1.29%	2.95%
11	1.40%	1.47%	0.60%	0.00%	−0.82%	0.85%
4	12.80%	7.49%	15.30%	−5.30%	2.55%	-7.83%
5						
9	2.10%	3.41%	2.90%	1.30%	0.78%	0.52%
7	2.60%	0.33%	3.60%	−2.20%	1.00%	−3.24%
6	6.90%	1.80%	5.90%	−5.10%	−1.08%	−4.05%
10						
8	2.10%	0.60%	0.50%	−1.50%	−1.65%	0.12%
17	0.30%	0.00%	0.00%	−0.30%	−0.23%	−0.03%
14*	2.40%	0.40%	0.00%	−2.00%	0.00%	0.00%
18*	0.30%	0.07%	0.00%	−0.20%	0.00%	0.00%
12*	0.80%	0.00%	0.00%	−0.80%	0.00%	0.00%
mean	97.00%	100.00%	98.10%			

The table shows the comparison of the predictions between both methods as well as with reference European frequencies from Jiang et al. 2012 (column 2), which is the source of the haplotype numbers (column 1). KIR*IMP’s haplotype frequencies for the 1496 GoNL haplotypes are in column 3; some haplotypes are combined, as the haplotype numbers distinguish KIR2DS4 alleles. Column 4 contains frequencies for EM-reduced KIR probe interpretation (KPI) haplotype predictions Column 5 compares KIR*IMP frequencies with the reference, as column 6 does for EM predictions. Finally, column 7 compares the frequencies of KIR*IMP and the EM-reduced predictions. Haplotypes with a predicted frequency of 0 in both KIR*IMP and KPI are not shown. Haplotypes 14, 18, and 12 are in KIR*IMP’s set of reference haplotypes, but not KPI’s.

## Discussion

KPI was evaluated by Chen et al. as part of a larger effort (18). In a cohort of 72 individuals with ground truth determined by LinkSeq qPCR, Chen et al. report six mismatches in one sample (possibly swapped), and apart from this 95.8% accuracy for *KIR2DS3* and 100% accuracy for the 15 other genes. As they note, it is now possible to interpret HLA binding alleles and the presence/absence of all their KIR receptors from short-read high-throughput sequencing, and this combination is a valuable advancement for research and medicine. Indeed, they compare KPI favorably with respect to clinical accreditation standards.

The findings by Chen et al. are consistent with the results of our synthetic test, whose accuracy was 100% for all genes. One drawback of the design of the synthetic test is that the haplotypes used in the test were also included in the MSA that was used to generate the per-gene probes. However, the main purposes of the synthetic tests were to test the application of the markers to short reads in a variety of genotypes and recover their original geno- and haplo-types; this is value-added compared with simply demonstrating sequences unique to a gene. The almost-perfect results of the Genentech and synthetic experiments, along with a GoNL results that had a 99.6% family-consistency rate and in line with expected frequencies, provide evidence that KPI’s gene predictions are very accurate.

The evidence also suggests that KPI’s haplotype results are accurate, although often ambiguous: the accuracy in the synthetic test was 100% and the GoNL family-consistency was 99.6, and the predictions allow EM predictions that align with the expected population frequency from the literature.

KIR*IMP’s haplotype frequency estimations differ from expectations in some areas. The evidence from comparisons with frequency reports from Jiang et al. 2012 (**Table 5**, column 5) suggest KIR*IMP overestimated cA01~tA01 (haplotype numbers 1 and 2) and underreported haplotypes containing cB01 or cB02 centromeric regions combined with the tA01 telomeric region (cB01~tA01 and cB02~tA01) in the GoNL cohort. This discrepancy can also be seen in the predicted genotype frequencies, where KIR*IMP relatively under calls the presence of *KIR2DS2*, *KIR2DL2*, and *KIR2DS3* by ~20% and *KIR2DL5* by ~10% compared with KPI and the historical European frequencies from Allele Frequency Net database (**Table 5**, column 6); all four of those genes are in cB01, and *KIR2DS2* and *KIR2DL2* are also in cB02. GoNL genotyping was done on the Immunochip, which is the best option according to the KIR*IMP manuscript. With that chip, they report accuracies of 100% for *KIR2DS2*, 98% for *KIR2DL2*, 82% for *KIR2DL5*, 81% for *KIR2DS3*, and 95% for *KIR2DS5* in their Norwegian-German validation cohort. Although the family consistency rate is 100% for KIR*IMP and 96.6% for KPI, their haplotype-pair predictions only agree in 58% of individuals. Without ground truth available, without any reason to expect this cohort to deviate from expectations, and considering KIR*IMP’s self-reported accuracy, the evidence suggests that KPI’s predictions are more accurate than KIR*IMP’s in this cohort and specifically that KIR*IMP under called the presence of genes *KIR2DS2*, *KIR2DL2*, *KIR2DS3*, *KIR2DL5* and haplotypes cB01~tA01 and cB02~tA01. As reviewed recently by Wright et al., this may be particularly relevant in the context of hematopoietic stem cell transplantation, where some case/control studies claim an important role for these regions (19). There are several potential reasons KIR*IMP’s predictions may be less accurate than KPI’s. The reference haplotypes used for marker discovery for KIR*IMP were defined by copy number genotyping and family relationships; KPI defined its haplotypes using a MSA of full haplotype sequences. KIR*IMP’s input is restricted to a few hundred single nucleotide polymorphisms, whereas KPI can use the entire genomic range of KIR sequences of length 25, which provides the potential for more information per marker and a broader base of markers. KIR*IMP uses a small number of SNPs to call one or more genes, whereas KPI uses dozens-to-thousands of 25mers to call a single gene, One of the steps of KIR*IMP’s workflow is to align and phase all the SNPs to one ‘A’ haplotype, which may be a limitation for genes not on that haplotype; all the gene and haplotypes we found to have lower accuracy rates are not located on the ‘A’ haplotype. KPI has no alignment or assembly steps. It is also important to note that the primary purpose for the comparison with KIR*IMP was not to evaluate the potential success of predicting KIR genes and haplotypes using SNPs vs sequence reads, but rather to compare the two algorithms. Although both algorithms predict the presence/absence of KIR genes and structural haplotypes, their solution domains are very different: microarray SNP panels vs raw genomic DNA reads. Both algorithms report the lowest accuracy rates for *KIR2DS3* and a ~10% lower frequency rate for *KIR2DS1* in GoNL compared with reference frequencies.

The 85% family consistency rate of the EM-reduced haplotype predictions suggest that KIR haplotype ambiguity cannot be accurately reduced at the individual level *via* expectation-maximization. However, since the EM-reduced haplotype frequencies are in line with references, it is possible the predictions might aggregate to population-level in a maximum-likelihood manner and therefore perhaps may still be useful for some population genetics purposes.

Traditional lab-based SSO presence/absence genotyping relies on a single short-sequence strategy, an approach that can be applied similarly to synthetic analysis of large amounts of WGS. In this virtual context, primer locations are not needed, and kmer searching is efficient and accurate at populations scales. To develop this synthetic SSO-like (kmer) library, we leveraged the information from a multiple sequence alignment of all full-length haplotypes that are available for this study. We believe this is a more accurate approach than using IPD-KIR reference alleles, because the IPD-KIR reference alleles do not require the haplotype location to be known. In addition, fusion alleles are assigned in IPD-KIR to one of the two parent genes, and therefore large sequences of some alleles are not really from the gene in which they are classified. We used 25 for our ‘k’ (i.e., sequence size) because BLAST searching indicated this to be a conservative minimum length needed to distinguish a small set of test markers to the KIR region. We did not experiment with any k size other than 25, since the choice gives a reasonable number of significant markers and their lack of off-KIR hits as tested in the GoNL population WGS confirms the effectiveness as gene/intergenic markers. The only fundamental benefit to shorter markers would be in the case when there were no longer markers; however, 25mer markers were found for every gene. The only fundamental benefit to longer markers would be if the markers were not unique to the region; however, all the markers are unique to their region. Many of the 25mers overlap each other, effectively simulating a single longer marker. Similar to the reasoning about probe length, probe mismatches would only need to be relaxed if a locus did not have any markers. Since at least one marker was discovered for each gene, mismatches did not need to be incorporated. Having thus obtained the region markers, we then used the most common (‘peak’) hit count from each gene/intergene’s library of sequences to make the PA genotype calls (**Supplemental Figure 1**). This adds a certain amount of allelic flexibility in the algorithm because the ultimate call is an average of all the markers for that gene; if some markers miscall, the overall call for the gene will be unaffected if the majority of the other markers are accurate. Since KPI decomposes the genetic information into 25mers, it works with any collection of DNA reads, as long as the KIR region is included. It works with fasta, fastq, single, paired, short, and long reads. Since the markers are not unique to exons, it will not work with cDNA or exon only reads.

One limitation of the method is that the markers do not mark DNA segments longer than one gene. Perhaps this is primarily due to the frequent recombination between haplotypes. Although recombination has been reported in multiple loci, the hotspot in between the centromeric and telomeric regions is particularly strong, and, in general, any pairing of the two can be expected. We evaluated single markers for haplotypes, but we did not find any. This is particularly relevant in light of the observation that haplotyping from genotypes seems to have limited accuracy under maximum likelihood assumptions (**Table 5**). It is possible that an algorithm that uses applies multiple markers in a hierarchical or combinational manner may be more successful. For future work, we plan to further evaluate *KIR2DS3* (95.8% accuracy in the Chen et al. evaluation) and evaluate the genotyping in diverse populations. It is possible that population robustness is a weakness of the method, although the fact that almost half of individuals in the discovery cohort are African or African American provides some optimism.

For the WGS data set, KPI averaged less than 1 h of computing time per individual, with 32 cores of CPU and 32G RAM. The majority of the time is spent using KMC 3 to build the kmer database. kmer counting is an active area of research. Since KPI can easily be altered to use any such application, it has potential for future efficiency improvements.

The markers discovered in this study were enabled by a full-haplotype MSA, as described recently by Roe et al. (11). That manuscript makes observations about the composition and order of sequences within KIR haplotypes, and it reports the implications for our understanding of the relationship between haplotypes, loci, and genes. Here, we have leveraged that basic understanding for practical use by developing a free and open interpretation application, evaluating a SNP interpretation algorithm, and contributing KIR interpretation to an important population genetics resource for Netherlands and many types of human genetic researchers. We described how the gene markers were discovered from the MSA, and we demonstrated their use to predict genes and haplotype-pairs at high accuracy (97%+) with population scale from any kind of sequence data that includes the full KIR region, including WGS. It was tested on synthetic ground-truth sequences and a large cohort of family WGS. In addition, we compared our algorithm to the leading SNP-based interpretation algorithm. KPI is free software with a GPL3 license and is implemented as a Nextflow workflow backed with an optional Docker environment. It is available at https://github.com/droeatumn/kpi.

## Author’s Note

This manuscript has been released as a pre-print at bioRxiv with the same title and authors (20).

## Data Availability Statement

The datasets presented in this study can be found in online repositories. The names of the repository/repositories and accession number(s) can be found in the article/**Supplementary Material**.

## Ethics Statement

The studies involving human participants were reviewed and approved by University of Minnesota. Written informed consent for participation was not required for this study in accordance with the national legislation and the institutional requirements.

## Author Contributions

DR and RK designed the experiments and wrote the manuscript. DR conducted the experiments. All authors contributed to the article and approved the submitted version.

## Conflict of Interest

The authors declare that the research was conducted in the absence of any commercial or financial relationships that could be construed as a potential conflict of interest.

## Supplementary Material

Supplemental Figure 1 Algorithm details. Data sheet 3 is a Microsoft Word document containing the commands to query the markers per genome and details as to how each region was genotyped.

Supplemental Data Sheet 1 Zipped text file containing the marker DNA sequences of length 25. The gene markers (column 2) are unique to their label (column 1), but the intergene and haplotype sequences are not.

Supplemental Data Sheet 2 Raw reads from synthetic evaluation, whose results are in **Table 2**. Each of the six simulated individuals have a pair of fastq files for each parental haplotype.

Supplemental Table 1 Individual gene and haplotype-pair predictions. Columns A and B contain the family name and relationship. Column C contains KPI’s haplotype-pair predictions, represented by a haplotype list. Each pair is separated with a ‘|’. For example, ‘cA01~tA01+ cA01~tB01_2DS5|cA01~tB01_2DS5+cA01~tB05’ means the prediction is either haplotype-pairs cA01~tA01 and cA01~tB01_2DS5 or haplotype-pairs cA01~tB01_2DS5 and cA01~tB05. The haplotype list in column C represents all possible haplotype-pairs fitting the presence-absence genotypes. Column D is a count of the number of haplotype-pair predictions in column C’s haplotype list. Column E is the haplotype list in column C reduced by family relationships, and column F indicates whether or not it is different from the original haplotype list in column C. Column G is the original haplotype list in column C reduced by EM; its results should not be used as they have limited accuracy. Columns H-W are the gene presence-absence predictions.
