# Supplementary material for: Accurate and Efficient KIR Gene and Haplotype Inference From Genome Sequencing Reads With Novel K-mer Signatures
Source: Front Immunol. 2020 Nov 26;11:583013. doi: 10.3389/fimmu.2020.583013 (PMC7727328; doi:10.3389/fimmu.2020.583013)
Supplement: Supplemental Figure 1 — Algorithm details. Data sheet 3 is a Microsoft Word document containing the commands to query the markers per genome and details as to how each region was genotyped. [file DataSheet_3.docx]

Algorithm details

**kmer marker discovery**

KPI calls a presence/absence per by aggregating the presence/absence genotype of many small (25mer) probes specific for that gene. This is the command to discover the 25mer probes for a gene, e.g., *KIR2DL1*

*kmc -k25 -ci72 -cx72 -fm ./2DL1/2DL1.fasta intersect/2DL1 intersect*

*kmc_tools complex intersect/./2DL1_cmd.txt*

*kmc_dump intersect/./2DL1_unique intersect/./2DL1_intersect.txt*

Once the all probes have been discovered, a KMC database was made for them and their reverse complements

*kmc -v -k25 -ci1 -fa intersect_union-All_v1_wRc.fasta intersect_union-All_v1_wRc work > intersect_union-All_v1_wRc_err.txt*

**Individual kmer genotyping**

To genotype a specific individual, a KMC 25mer database was generated from their raw sequences, e.g., 52b. gonl-52b-cmd.txt has six lines, each with the full path to a FASTQ file.

*kmc -k25 -ci2 -fq @./gonl-52b-cmd.txt ./gonl-52b work*

Each probes hit count was obtained by interesting the probe database with the individual’s genotype database. The output is a collection of marker names and a count for each marker. e.g., 134a

*kmc_tools -hp simple .//gonl-134a intersect_union-All_v1_wRc intersect gonl-134a -ocleft*

*kmc_tools dump gonl-134a gonl-134a.txt*

**Generating gene presence/absence via probe marker counts**

Presence/absence calls for each gene region were made by the peak hit count per region in one individual, with counts of 1 or 2 errors and therefore set to a count of 0. The peak is the most common hit count for all markers in that region. If the most common hit count is zero, the region is called absent; if the hit count is greater than zero, the region is called present.

Figure 1 shows *KIR3DL1*’s 25mer hit distribution for a ‘present’ genotype in one individual. The most common occurrence (or peak of the chart) is 102 25mers that hit 11 times. Since the peak is greater than 0 hits, *KIR3DL1* is called present.

Figure 1. Example hit distribution for a ‘present’ genotype. The x axis shows the number of hits in the genome. The y axis shows the number of 25mers with that hit count.

Figure 2 shows *KIR3DS1*’s 25mer hit distribution for an ‘absent’ genotype in one individual. The most common occurrence (or peak of the chart) is 1319 25mers that have 0 hits. Since the peak is 0 hits, *KIR3DS1* is called absent.

Figure 2. Example hit distribution for a ‘absent’ genotype. The x axis shows the number of hits in the genome. The y axis shows the number of 25mers with that hit count.
